# Rescuing AAV gene transfer from neutralizing antibodies with an IgG-degrading enzyme

**DOI:** 10.1172/jci.insight.139881

**Published:** 2020-09-17

**Authors:** Zachary C. Elmore, Daniel K. Oh, Katherine E. Simon, Marco M. Fanous, Aravind Asokan

**Affiliations:** 1 Department of Surgery and; 2 Department of Molecular Genetics and Microbiology, Duke University School of Medicine, Durham, North Carolina, USA.; 3 Department of Biomedical Engineering, Pratt School of Engineering, and; 4 Regeneration Next, Duke University, Durham, North Carolina, USA.

**Keywords:** Immunology, Therapeutics, Gene therapy

## Abstract

Preexisting humoral immunity to recombinant adeno-associated virus (AAV) vectors restricts the treatable patient population and efficacy of human gene therapies. Approaches to clear neutralizing antibodies (NAbs), such as plasmapheresis and immunosuppression, are either ineffective or cause undesirable side effects. Here, we describe a clinically relevant strategy to rapidly and transiently degrade NAbs before AAV administration using an IgG-degrading enzyme (IdeZ). We demonstrate that recombinant IdeZ efficiently cleaved IgG in dog, monkey, and human antisera. Prophylactically administered IdeZ cleaved circulating human IgG in mice and prevented AAV neutralization in vivo. In macaques, a single intravenous dose of IdeZ rescued AAV transduction by transiently reversing seropositivity. Importantly, IdeZ efficiently cleaved NAbs and rescued AAV transduction in mice passively immunized with individual human donor sera representing a diverse population. Our antibody clearance approach presents a potentially new paradigm for expanding the prospective patient cohort and improving efficacy of AAV gene therapy.

## Abstract 2

An antibody clearance strategy to rapidly and transiently degrade neutralizing antibodies using an IgG degrading enzyme prior to AAV administration could potentially improve the efficacy of AAV gene therapy.

## Graphical abstract

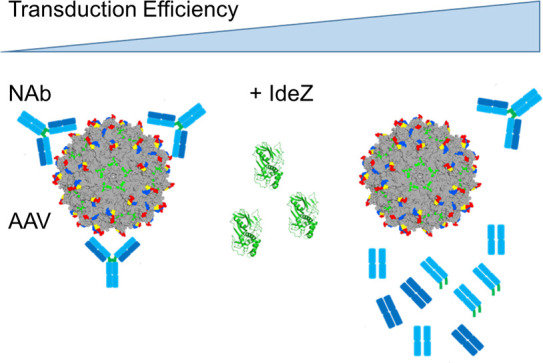

## Introduction

Human gene therapy using recombinant adeno-associated virus (AAV) vectors continues to advance steadily as a treatment paradigm for rare, monogenic disorders. This is highlighted by the recent FDA approval and clinical success of Zolgensma, an intravenously dosed AAV vector delivering a functional copy of the *SMN1* gene in children with spinal muscular atrophy (1). Further, the list of systemically dosed AAV-based gene therapies for rare disorders, such as hemophilia A and B, Duchenne muscular dystrophy, X-linked myotubularin myopathy, and Pompe disease, continues to grow (2, 3). These promising clinical examples have concurrently highlighted important challenges that include manufacturing needs, patient recruitment, and the potential for toxicity at high AAV doses. One such challenge that limits the recruitment of patients for gene therapy clinical trials and adversely affects the efficacy of AAV gene therapy is the prevalence of preexisting neutralizing antibodies (NAbs) against AAV capsids in the human population. Such NAbs arise because of natural infection or cross-reactivity between different AAV serotypes (4–7). NAbs can mitigate AAV infection through multiple mechanisms by (a) binding to AAV capsids and blocking critical steps in transduction such as cell surface attachment and uptake, endosomal escape, productive trafficking to the nucleus, or uncoating and (b) promoting AAV opsonization by phagocytic cells, thereby mediating their rapid clearance from the circulation. Multiple preclinical studies in different animal models have demonstrated that preexisting NAbs impede systemic gene transfer by AAV vectors (8–11).

In humans, serological studies reveal a high prevalence of NAbs in the worldwide population, with about 67% of people having antibodies against AAV1, 72% against AAV2, and approximately 40% against AAV serotypes 5 through 9 (4, 12–14). Because of this high NAb seroprevalence, screening for AAV antisera through in vitro NAb assays or ELISA is commonplace in AAV gene therapy trials, and exclusion criteria can render upward of 50% of patients ineligible for treatment or admission into clinical trials (15, 16). Furthermore, vector immunogenicity represents a major challenge in readministration of AAV vectors. High-titer NAbs are produced following AAV vector administration, thereby preventing prospective AAV redosing (6, 17). This severely limits long-term gene therapy success in (a) patients in the low-dose AAV cohort, (b) pediatric patients who will experience tissue growth and proliferation leading to vector genome dilution and potential reversal of symptoms with age, and (c) patients with degenerative disorders that might require multiple AAV treatments to prevent tissue loss and subtherapeutic transgene expression levels. Taken together, NAbs present a significant barrier to the broad application of AAV in the clinic.

**Figure 1:**
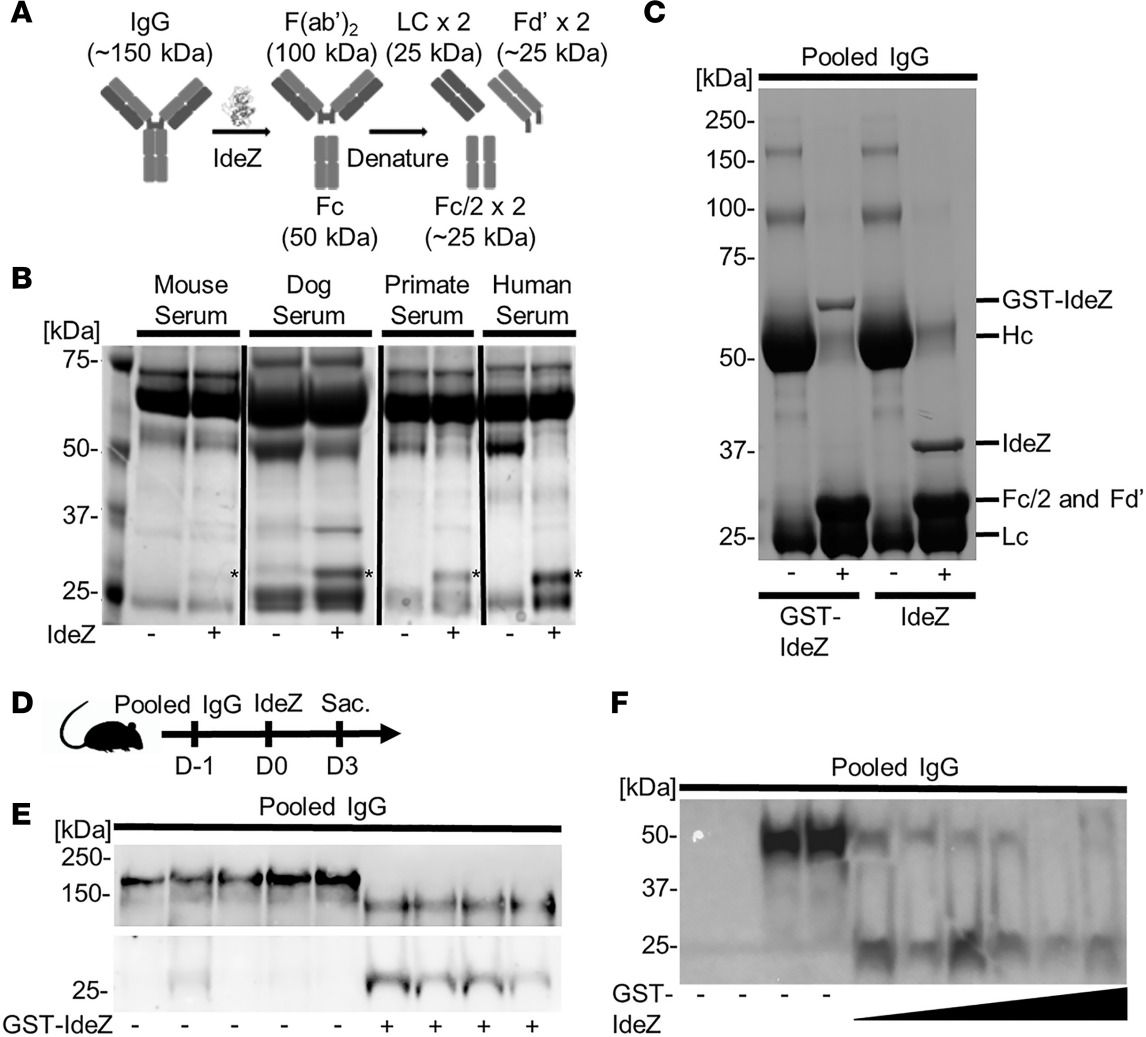
IdeZ cleaves serum antibodies from multiple species. (**A**) Schematic outlining IdeZ cleavage of IgG below the hinge region yielding multiple F(ab′)_2_ and Fc fragments after reduction. (**B**) Serum samples from mouse, dog, primate, and human untreated (-) or treated (+) with recombinant IdeZ and analyzed by SDS-PAGE under reducing conditions. Gels were then stained with Coomassie blue. * indicates digested Fc/2 and Fd′ fragments. (**C**) Pooled human IgG untreated (-) or treated (+) with recombinant glutathione-*S*-transferase–IdeZ (GST-IdeZ) or commercial standard IdeZ and analyzed by SDS-PAGE under reducing conditions. Gels were then stained with Coomassie blue. IgG was cleaved by GST-IdeZ and IdeZ into multiple fragments as indicated. (**D**) Experimental timeline of in vivo GST-IdeZ dose optimization experiment. Mice were injected with pooled human IgG followed 24 hours later with no injection or injection with 3 different doses of GST-IdeZ. Blood serum samples were collected 72 hours post GST-IdeZ. Sac., sacrifice followed by tissue harvest. (**E**) Mice were injected IP first with pooled human IgG, followed by IV injection with PBS 24 hours later (-) or recombinant GST-IdeZ (1 mg/kg) (+). Blood samples were taken 72 hours after injection and analyzed by SDS-PAGE under reducing conditions with immunoblotting. IgG was probed with Fab- and Fc-specific antibodies. (**F**) Mice were injected IP first with pooled human IgG, followed by IV injection with PBS 24 hours later (-) or recombinant GST-IdeZ at 3 doses (0.25 mg/kg, 1 mg/kg, and 2.5 mg/kg) (+). Blood samples were taken 72 hours after injection and analyzed by SDS-PAGE under reducing conditions with immunoblotting. Human IgG was probed with an Fc-specific antibody. See complete unedited blots in the supplemental material.

Strategies that are currently being evaluated to circumvent preexisting humoral immunity to AAV vectors are early in development, ineffective, or prone to causing undesirable side effects. These include the engineering of new AAV variants with reduced NAb recognition (18, 19), plasmapheresis or immunoadsorption to reduce the overall levels of circulating antibodies in patient serum before AAV administration (20–23), use of capsid decoys (24), or immunosuppression to decrease the B cell population and consequently antibody levels in general (25, 26). While these approaches have demonstrated varying success and efficiency in addressing the problem of circulating antibodies and remain under evaluation, a one-solution-fits-all approach that resolves this challenge is unlikely. Pertinent to this, a promising and clinically validated paradigm for mitigating the effects of deleterious (auto)antibodies is the use of IgG-specific proteases (27–30). In particular, the extracellular enzyme IdeS, derived from *Streptococcus pyogenes*, is a 35 kDa cysteine protease that specifically cleaves IgG at the lower hinge region, generating 1 F(ab′)_2_ fragment and 1 homodimeric Fc fragment (31–34) (Figure 1A). IdeZ, a homolog of IdeS, was identified and characterized in *S*. *equi* ssp. *zooepidemicus* and shown to efficiently cleave IgG in a similar manner to IdeS (35, 36). Here, we evaluate the ability of IdeZ to mitigate the effect of preexisting anti-AAV NAbs in mice passively immunized with human antisera and in nonhuman primates. First, we demonstrate the ability of IdeZ to cleave antibodies in sera derived from multiple species. Next, we show that IdeZ can rescue AAV gene transfer in the presence of circulating human IgG in mice and natural humoral immunity in nonhuman primates. In addition, we demonstrate that gene transfer to the liver and heart is also rescued in mice passively immunized with individual human antisera.

## Results

### IdeZ shows robust ability to cleave antibodies in sera from multiple species.

We first demonstrated that IdeZ efficiently cleaves antibodies in canine, nonhuman primate, and human sera but not mouse serum samples in vitro (Figure 1B). The latter observation is corroborated by known mutations in the hinge region of mouse IgG compared with other species that render the latter resistant to IdeZ-mediated degradation (35, 36). IdeZ also effectively cleaved human IgG into heavy chain, light chain, and Fc fragments in vitro (Figure 1C). Specifically, IdeZ cleavage generates 1 F(ab′)_2_ fragment and 1 homodimeric Fc fragment held together by noncovalent interactions. Under denaturing conditions such as SDS-PAGE, the F(ab′)_2_ fragment dissociates into 2 light chain and 2 Fd′ regions followed by the Fc region dissociating into 2 Fc/2 regions (31–34). Next, we confirmed the potency of research-grade, recombinant GST-tagged IdeZ produced in *E. coli* for dosing in vivo (Figure 1D). Mice were first passively immunized with pooled IgG injected intraperitoneally (IP), followed by a single intravenous (IV) injection of IdeZ confirming efficient cleavage into Fab and Fc fragments as determined by Western blotting (Figure 1E). Further, as shown in Figure 1F using an anti-Fc antibody, we observed a dose-dependent effect in IgG degradation, with near total clearance at 2.5 mg/kg of IdeZ after day 2 postadministration. The Western blot showing the total IgG fragments in sera post–IdeZ treatment corroborated the observation that effective clearance of circulating antibodies occurred within 1 or 2 days post–IdeZ administration (Supplemental Figure 1; supplemental material available online with this article; https://doi.org/10.1172/jci.insight.139881DS1). Furthermore, IdeZ effectively mitigated human IgG–mediated neutralization of AAV8- and AAV9-Luc transduction in vitro (Supplemental Figure 2), leading us to investigate the efficacy of IdeZ treatment on AAV gene transfer efficiency in the presence of neutralizing antisera in vivo.

### IdeZ rescues AAV liver gene transfer in mice and macaques.

**Figure 2:**
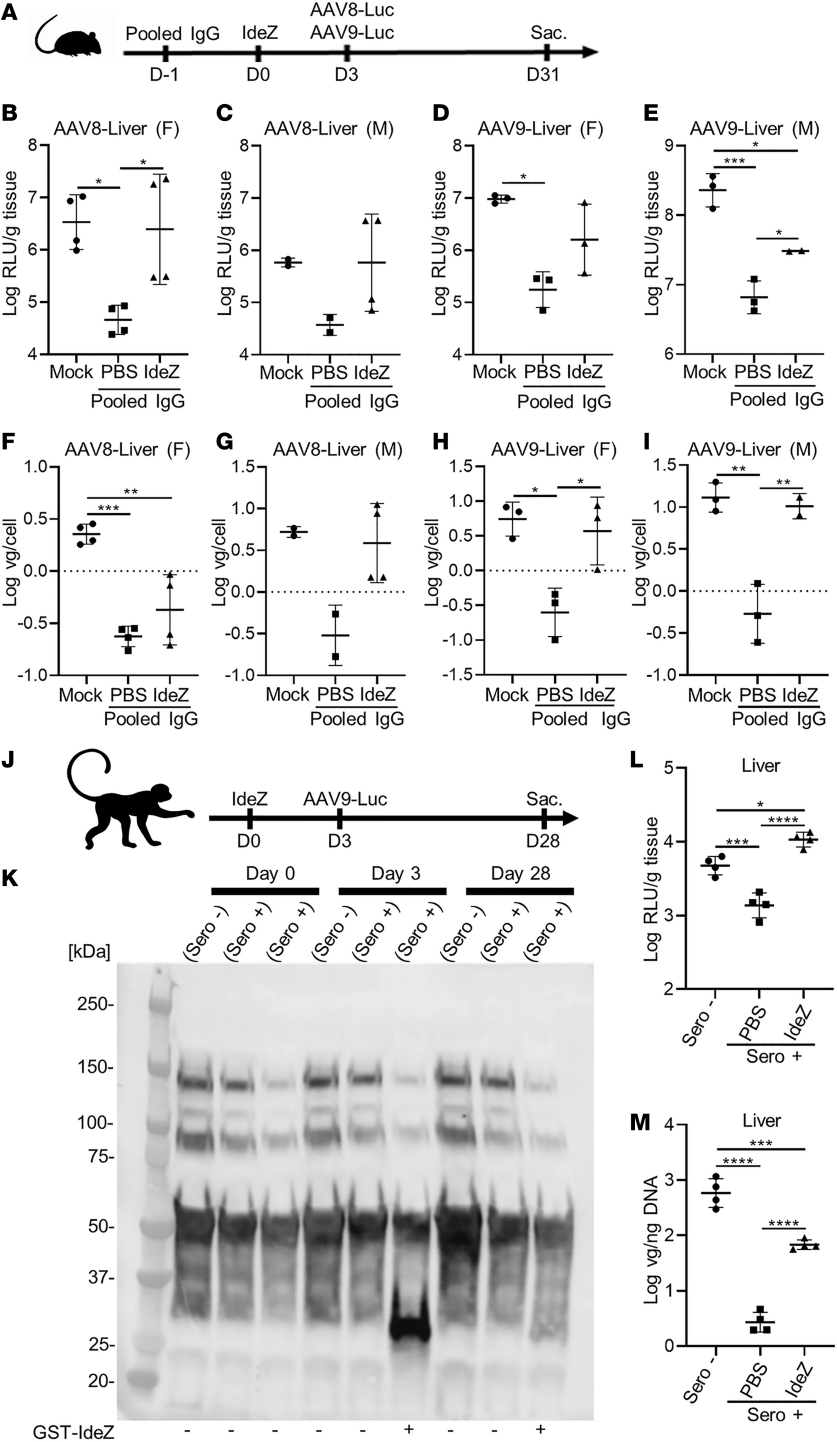
IdeZ rescues AAV8 and AAV9 liver transduction in passively immunized mice and cynomolgus macaques with preexisting NAbs. (**A**) Experimental timeline of IgG, IdeZ, and AAV8- or AAV9-Luc injections. Sac., sacrifice followed by tissue harvest. Mice were injected IP with pooled human IgG. The same mice were injected IV 24 hours later with PBS or recombinant GST-IdeZ (2.5 mg/kg). AAV8-Luc or AAV9-Luc was injected 72 hours post–IdeZ treatment at a dose of 1 × 10^13^ vg/kg. Luciferase transgene expression levels were analyzed 4 weeks postinjection in the liver: AAV8 (**B** and **C**) and AAV9 (**D** and **E**). Luciferase expression levels were normalized for total tissue protein concentration and represented as log relative luminescence units per gram of tissue (log RLU/g tissue). Each dot represents the average of a technical duplicate from a single animal. Biodistribution of AAV8- and AAV9-Luc vector genomes in the liver: AAV8 (**F** and **G**) and AAV9 (**H** and **I**). Vector genome copy numbers per cell were calculated by normalizing Luc copy numbers to copies of the lamin B2 housekeeping gene and represented as log vg/cell. Each dot represents a technical duplicate from a single animal, and the dash represents the mean value. (F, female; M, male.) (**J**) Schematic demonstrating experimental timeline of IdeZ and AAV9-Luc injections in nonhuman primates (NHPs). AAV9-seropositive NHP M16558 (*n* = 1) was administered IdeZ (0.5 mg/kg) via IV bolus injection on day 0. AAV9-Luc was administered via IV bolus injection 72 hours post–IdeZ injection at a dose of 5 × 10^12^ vg/kg. (**K**) NHP serum samples were analyzed by SDS-PAGE under reducing conditions and probed with Fc-specific antibodies. (**L**) Luciferase transgene expression levels were analyzed 4 weeks postinjection in the livers of NHPs. Luciferase expression levels were normalized for total tissue protein concentration and represented as log relative luminescence units per gram of tissue. Each dot represents a single experiment of an individual liver lobe from a single animal. (**M**) Biodistribution of AAV9-Luc vector genomes in the livers of NHPs. Vector genome copy numbers per nanogram of total extracted DNA were calculated and represented as log vg/ng DNA. Each dot represents a technical duplicate experiment of individual liver slices from a single animal, and the dash represents the mean value. Significance was determined by 1-way ANOVA with Tukey’s posttest. *P < 0.05, **P < 0.01, ***P < 0.001, ****P < 0.0001.

Based on these results, we evaluated the ability of prophylactically dosing IdeZ in mice passively immunized with pooled human IgG to rescue AAV transduction in vivo. Briefly, animals of either sex were first injected IP with pooled human IgG (8 mg) on day –1, with a single dose of IdeZ (2.5 mg/kg) through the tail vein on day 0 and an IV dose of AAV8 or AAV9 vectors (1 × 10^13^ vector genome copies/kg [vg/kg]) packaging a chicken β-actin promoter–driven (CBA promoter–driven) luciferase transgene on day 3 (Figure 2A). Naive mice showed different levels of AAV8- and AAV9-mediated luciferase expression in the liver (Figure 2, B–E). In mice passively immunized with pooled human IgG, luciferase expression in the liver was decreased by 10- to 100-fold because of the presence of anti-AAV NAbs. In contrast, we observed rescue from AAV neutralization in IdeZ-treated animals, with partial to complete rescue of liver luciferase expression levels. These observations were corroborated by vector genome copy numbers, which corresponded with transgene expression in general, although we observed sex-specific differences (Figure 2, F–I). Notably, despite restoration of AAV copy numbers in the male liver, expression was not fully restored, implying that other non–NAb-related factors might be involved in controlling liver expression (Figure 2, C and E). While these aspects warrant further investigation and dose optimization, these observations support that prophylactically administered IdeZ can prevent AAV neutralization and restore liver transduction in an AAV serotype–independent manner.

Next, we sought to evaluate whether IdeZ was effective in nonhuman primates. We first screened male cynomolgus macaques for anti-AAV antibodies using a NAb assay to identify seropositive and seronegative animals (Supplemental Figure 3). Animal M16561 served as the naive seronegative control, while the seropositive animals M16556 and M16558 were dosed on day 0 with IV PBS or a single IV bolus dose of IdeZ (0.5 mg/kg), respectively. On day 3 post–IdeZ treatment, all 3 animals were injected with a dose of AAV9 vectors packaging the luciferase transgene (5 × 10^12^ vg/kg) (Figure 2J). Evaluation of serum IgG levels at days 0, 3, and 31 post–IdeZ treatment revealed selective cleavage and clearance at day 3. In addition, serum IgG levels were fully restored to normal levels by day 31, corroborating the transient effect of IdeZ activity (Figure 2K). Upon sacrifice at day 30, we observed an approximately 1 log order decrease in luciferase gene expression and a disproportionate (~2 logs) decrease in vector genome copy number in the liver (Figure 2, L and M). Importantly, IdeZ treatment restored AAV luciferase gene expression levels and partially restored vector genome copy numbers in the liver. Further, these results also mirrored the observations in the liver of male mice injected with human IgG. Although the number of nonhuman primates in the current study is low, the above results underscore the ability to translate the applicability of IdeZ in clearing IgG across multiple species. It should be emphasized that the NHPs used in this study were not passively immunized but demonstrated moderate preexisting NAb titers against AAV arising from natural exposure. However, it is also important to note that the IdeZ dose used in our NHP study is 5-fold lower than that used in passive immunization studies in mice (0.5 mg/kg vs. 2.5 mg/kg), emphasizing a need for future dose optimization studies in large animal models.

### IdeZ rescues AAV liver gene transfer in mice passively immunized with individual human sera.

**Figure 3:**
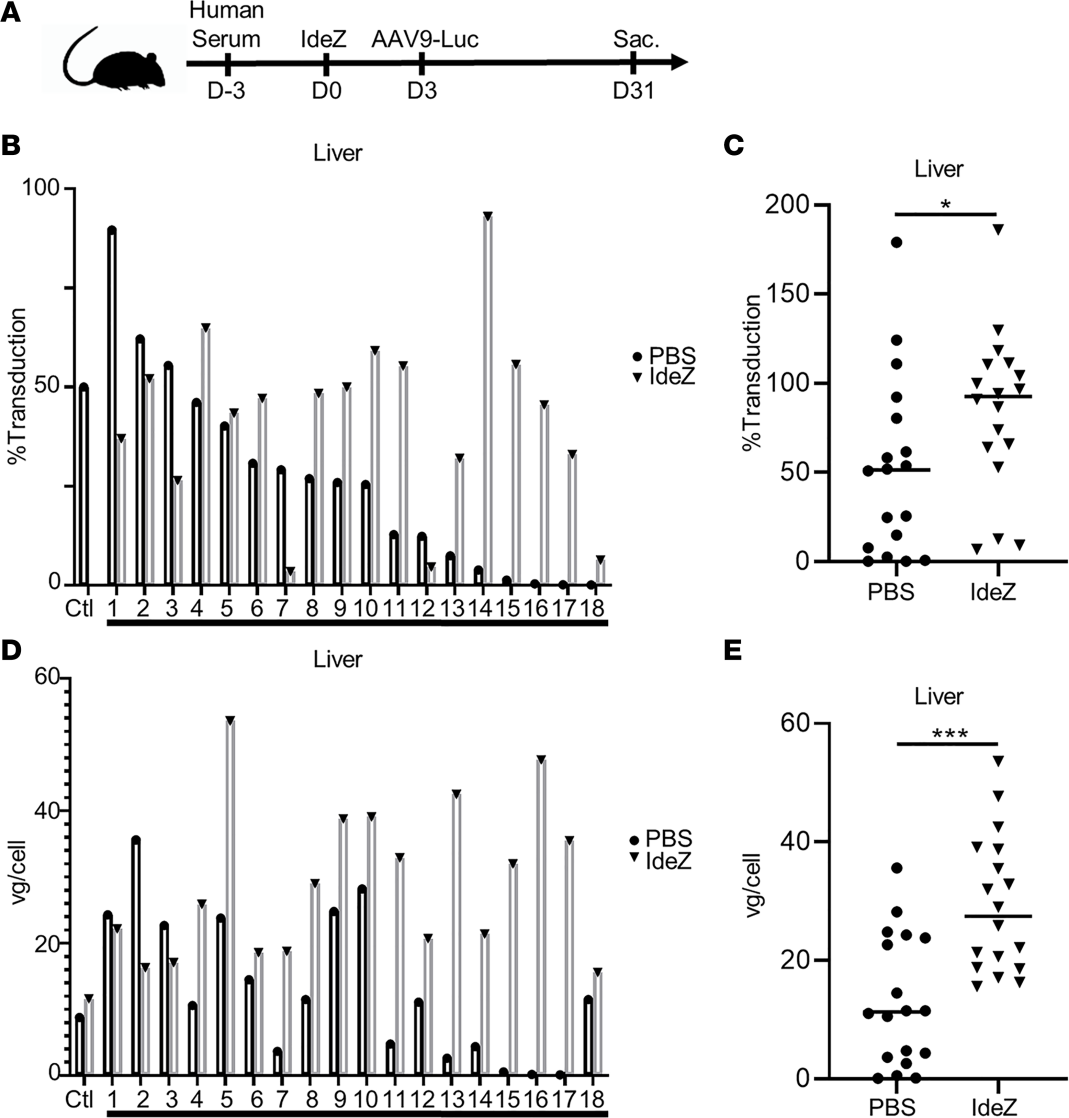
IdeZ rescues AAV9 liver transduction in mice passively immunized with individual human sera. (**A**) Schematic demonstrating experimental timeline of human serum, IdeZ, and AAV9-Luc injections. Eighteen human serum samples were tested for their ability to neutralize AAV9 transduction in the liver. Two mice per human serum sample were used for the study, and both mice were injected IP with human serum. Mice were then injected IV 72 hours later with PBS (black bars) or recombinant GST-IdeZ (2.5 mg/kg, gray bars) and subsequently injected IV 72 hours post–IdeZ treatment with AAV9-Luc (1 × 10^13^ vg/kg). Liver transduction levels were analyzed 4 weeks postinjection. (**B**) Luciferase transgene expression levels were analyzed 4 weeks postinjection in the livers of passively immunized mice treated with PBS (black bar, black circle) or prophylactically with IdeZ (gray bar, black triangle). Transduction levels were normalized to nonimmunized mice that were injected with AAV9-Luc at the same dose and represented as percentage of control. Each bar represents the average of a technical duplicate from a single animal. (**C**) Relative liver transduction efficiency of AAV9-Luc in the entire cohort of mice immunized with human sera treated with PBS control (circle) or IdeZ (triangle). Biodistribution of AAV9 vector genomes in the liver for mice passively immunized with individual human serum samples (**D**) and the entire cohort (**E**). Vector genome copy numbers per cell were calculated based on normalization to copies of the lamin B2 housekeeping gene. Each bar represents the average of a technical duplicate from a single animal. Significance was determined by the nonparametric Mann-Whitney rank test. *P < 0.05, ***P < 0.001.

To further evaluate whether IdeZ can function effectively in a clinically relevant setting, we tested our approach in mice passively immunized with individual human donor sera representing a diverse population. Briefly, we obtained 18 human donor serum samples across a broad demographic and displaying varying levels of AAV neutralization as determined by NAb assay (Supplemental Figure 4). We then administered a single IP dose of donor serum in 2 animals each (total 18 cohorts), following which the first animal received an IV injection of PBS and the second, a single IV bolus dose of IdeZ (0.5 mg/kg). The control cohort comprised naive mice. All animals received an IV dose of AAV9-Luc vectors (1 × 10^13^ vg/kg), and luciferase gene expression was assessed in the liver and heart of the saline- versus IdeZ-treated cohorts (Figure 3A). As seen in Figure 3, B–E, the diversity of preexisting humoral immunity to AAV transduction is well represented by this small, yet diverse, panel of human serum samples (Supplemental Table 1). Notably, we observed restoration in liver luciferase expression levels in a number of animals (Figure 3, B and D). Complete restoration (100%) of liver expression to that of naive, nonimmunized control animals was observed in these animals regardless of NAb titer. Some outliers were also observed, where IdeZ treatment was only partially effective or adversely affected transduction. One possible explanation is that these mice might have high levels of preexisting immunity to IdeZ, although the impact of such on IdeZ activity is unclear. While these aspects warrant further investigation, we observed overall trends that support that IdeZ treatment can result in a statistically significant improvement in liver gene expression and copy number by clearing circulating antibodies (Figure 3, C and E). These results further underscore the potential for clinical translation with our approach.

### IdeZ-mediated rescue of AAV cardiac gene transfer efficiency provides additional insight into plausible neutralization mechanisms.

**Figure 4:**
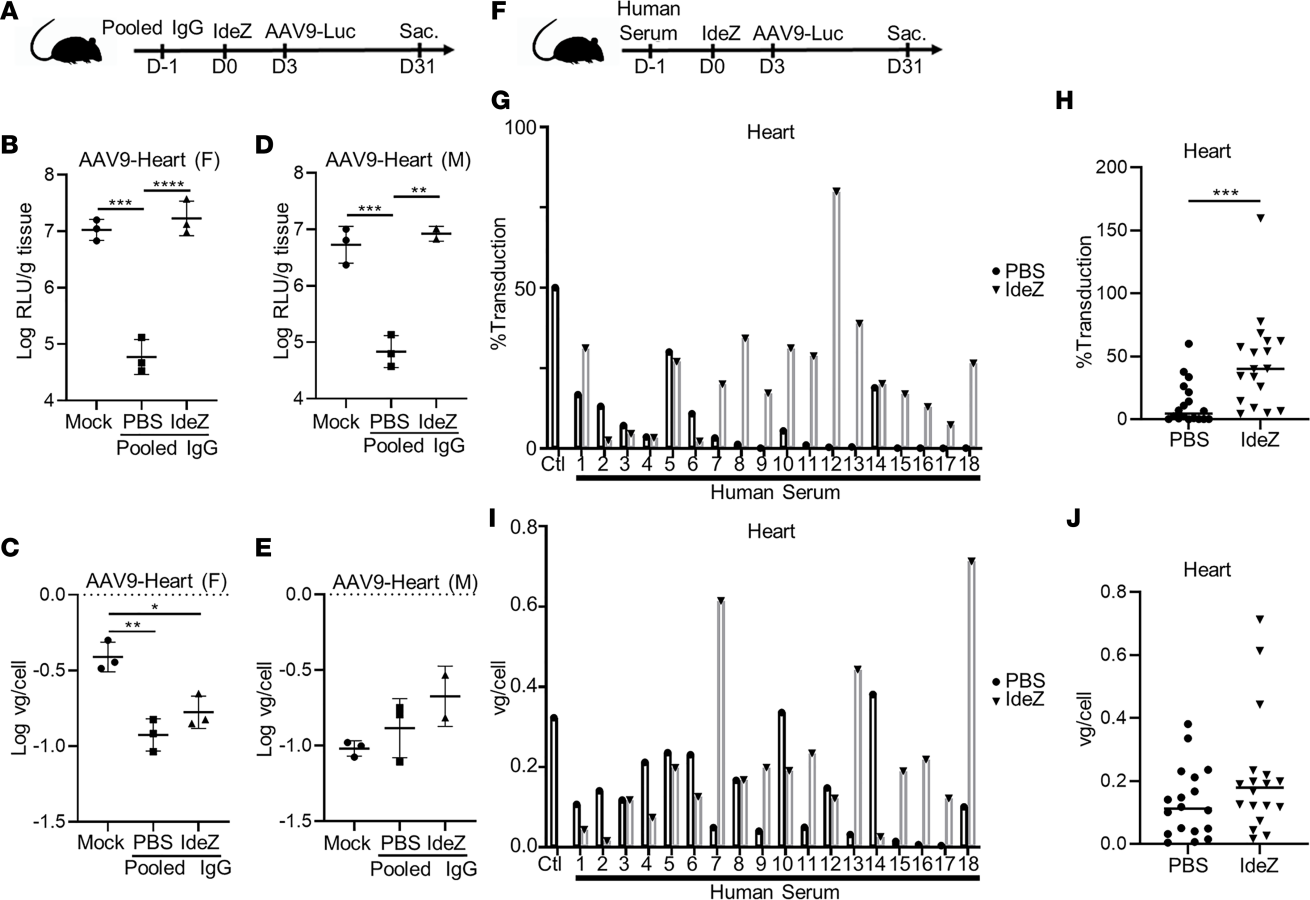
Impact of IdeZ treatment on AAV9 cardiac transduction in passively immunized mice. (**A**) Experimental timeline of pooled human IgG, IdeZ, and AAV9-Luc injections. Cardiac tissues were derived as outlined earlier in the liver experiment. (**B** and **D**) Luciferase transgene expression levels were analyzed 4 weeks postinjection in the heart. Luciferase expression levels were normalized for total tissue protein concentration and represented as log relative luminescence units per gram of tissue. Each dot represents the average of a technical duplicate from a single animal. (**C** and **E**) Biodistribution of AAV9-Luc vector genomes in the heart. Vector genome copy numbers per cell were calculated based on normalization to copies of the lamin B2 housekeeping gene. Each dot represents the average of a technical duplicate from a single animal. Significance was determined by 1-way ANOVA with Tukey’s posttest. *P < 0.05, **P < 0.01, ***P < 0.001, ****P < 0.0001. (**F**) Schematic demonstrating experimental timeline of human serum, IdeZ, and AAV9-Luc injections. Cardiac tissues were derived as outlined earlier in the liver experiment. (**G**) Luciferase transgene expression levels were analyzed 4 weeks postinjection in the hearts of passively immunized mice treated with PBS (black bar, black circle) or prophylactically with IdeZ (gray bar, black triangle). Transduction levels were normalized to nonimmunized mice that were injected with AAV9-Luc at the same dose and represented as percentage of control. Each bar represents the average of a technical duplicate from a single animal. (**H**) Relative cardiac transduction efficiency of AAV9-Luc in the entire cohort of mice immunized with human sera treated with PBS control (circle) or IdeZ (triangle). Biodistribution of AAV9 vector genomes in the heart for mice passively immunized with individual human serum samples (**I**) and the entire cohort (**J**). Vector genome copy numbers per cell were calculated based on normalization to copies of the lamin B2 housekeeping gene. Each bar represents the average of a technical duplicate from a single animal. Significance was determined by the nonparametric Mann-Whitney rank test. *P < 0.05, **P < 0.01, ***P < 0.001, ****P < 0.0001.

Concurrent to studies focused on restoring AAV gene transfer in the liver, we also analyzed the heart and observed striking differences. We assessed cardiac gene transfer in both mice passively immunized with pooled human IgG as well as individual human sera. Notably, although pooled human IgG decreased expression and IdeZ treatment restored cardiac luciferase expression levels to that of naive mice, changes in vector genome copy number upon IdeZ treatment were only partially rescued in females or statistically insignificant in males (Figure 4, A–E). These observations were further corroborated in mice passively immunized with individual human sera. In this regard, we first observed that neutralization of cardiac transduction by individual antisera did not mirror the patterns observed in the liver (Figure 3, B and D; and Figure 4, F, G, and I, black columns). Second, only partial rescue of AAV-mediated cardiac gene expression was observed in most animals. In addition, although we observed some increase in vector genome copy numbers within cardiac tissue, no specific correlation with luciferase expression patterns was noted (Figure 4, G and I). Assessment of overall rescue across the human sera–infused cohorts corroborated these trends (Figure 4, H and J). In particular, we observed a statistically significant rescue of cardiac gene transfer in cardiac luciferase expression but not vector genome copy numbers.

## Discussion

The IgG-degrading enzyme IdeS, also known as imlifidase, has shown promise in a clinical trial (ClinicalTrials.gov NCT02224820), permitting successful kidney transplantation in patients harboring donor-specific antibodies (37–39). Briefly, the latter study assessed the safety, immunogenicity, pharmacokinetics, and efficacy of imlifidase in an open-label, dose escalation study in highly sensitized patients with anti-HLA antibodies and chronic kidney disease. This approach represents a potential paradigm-shifting method to desensitize patients, who would otherwise not qualify to receive a lifesaving transplant. Thus, a clinical precedent for applying enzymatic IgG degradation to promote rapid and transient antibody clearance already exists. It is noteworthy to mention that other orthogonal methods to facilitate IgG clearance using soluble antibody-binding bacterial proteins (e.g., protein M, ref. 40), neonatal Fc receptor (FcRn) domains (41), and anti-FcRn antibodies such as rozanolixizumab (42), SYNT001 (43, 44), and so on have shown promise in the clinic as well. It is also important to note that immunosuppressive regimens have demonstrated promise in modulating AAV immunogenicity (26, 45–47) and could be used in parallel, providing a complementary approach to IdeS/IdeZ-mediated IgG degradation.

These approaches, however, have not been explored in the context of gene therapy to date to our knowledge. The antibody degradation/clearance approach described in the current study could broadly influence preclinical gene therapy studies in different large-animal models, currently encumbered by preexisting NAbs. For instance, preexisting humoral immunity against different AAV serotypes in macaques, dogs, and pigs has been described (8, 10). Based on our in vitro results, we postulate that IdeZ could potentially be applicable for evaluating AAV gene therapies in canine models of disease. These data combined with our observations in NHPs greatly expand the potential for preclinical AAV gene transfer studies but also provide a path toward safety and dose-finding studies using this approach in preclinical animal models. Additional studies to evaluate IdeZ dosing and kinetics of antibody clearance in such animal subjects with varying anti-AAV antibody titers is likely to help optimize this approach.

Another important advantage of the IdeZ approach is the potential for AAV serotype–independent rescue from antibody neutralization. Although this will require dose optimization studies with different natural and engineered AAV capsids, we postulate that the universality of our antibody clearance approach is likely to broadly complement AAV gene transfer studies. One possible caveat of this approach is that people may harbor antibodies against IdeZ similar to anti-IdeS antibodies described in previous studies (32, 48). In a dose escalation study (48), antibodies against IdeS were generated; however, it is not clear how anti-IdeS antibodies affect IdeS activity or immunogenicity upon retreatment in patients. However, animal studies whereby anti-IdeS antibodies are present do not prevent cleavage of IgG by IdeS (32). It is interesting to note that IdeZ, which is only approximately 68% identical in sequence similarity to IdeS, would likely degrade such antibodies as well. Furthermore, previous work (27) had patients immunosuppressed after transplantation, which was well after IdeS administration. It is important to note that these aspects will need to be thoroughly evaluated in subsequent studies using IdeS/IdeZ to rescue AAV gene transfer in the clinic. Another significant topic that warrants further evaluation is whether IdeZ treatment can enable vector redosing. In particular, IdeZ could provide an alternative solution in patients, where immunosuppression is infeasible or undesirable (25, 26, 47). Although we were unable to evaluate this in mice because of the inability of IdeZ to cleave mouse IgG, such studies should be feasible in NHPs or other animal models in the future. During the review of this manuscript, work was published highlighting the ability of imlifidase to rescue AAV liver transduction in passively immunized mice, NHPs with preexisting NAbs, as well as in NHPs predosed with AAV vectors (49). While corroborating the potential of IgG-degrading enzymes and their use for gene therapy applications, our study is differentiated in 4 key areas. First, our study uses a different IgG-degrading enzyme (IdeZ) that is only 68% similar to IdeS. Patients are unlikely to have neutralizing antibodies against IdeZ because of its originating from bacteria *S*. *equi* ssp. *zooepidemicus* isolated from horses. Second, we demonstrate the ability of IdeZ to rescue in vitro and in vivo transduction of 2 AAV serotypes, AAV8 and AAV9, expanding the scope of this approach. Third, our study highlights the effectiveness of IdeZ in rescuing AAV transduction of mice passively immunized with human sera (18 samples) from a diverse patient population. Fourth, our study expands the scope of gene therapy applications by evaluating rescue of AAV transduction in cardiac tissue in the presence of circulating NAbs. Taken together, from a clinical perspective, IdeZ has the potential to significantly influence the treatable patient population and improve the efficacy of AAV gene therapies.

## Methods

### Plasmid constructs and recombinant protein expression.

IdeZ DNA sequence from *S*. *equi* ssp. *zooepidemicus* lacking the N-terminal signal sequence was synthesized and cloned into the pGEX-6P-3 expression vector using BamHI and SalI restriction sites (GenScript). *E*. *coli* strain BL21 star (DE3, Thermo Fisher Scientific) was transformed with recombinant IdeZ pGEX-6P-3 plasmid. A single colony was inoculated into Terrific Broth (TB) medium containing ampicillin; culture was incubated in 37°C at 200 rpm and then induced with isopropyl β-d-1-thiogalactopyranoside (IPTG). Recombinant BL21 cells stored in glycerol were inoculated into TB medium containing ampicillin and cultured at 37°C. When the OD_600_ reached about 4, the cell culture was induced with IPTG at 37°C for 4 hours. Cells were harvested by centrifugation. Cell pellets were resuspended with GST lysis buffer followed by sonication. The supernatant after centrifugation was kept for future purification. Target protein was obtained by 2-step purification using a GST column and Superdex 200 column (GE Life Sciences). Target protein was sterilized by 0.22 μm filter before being stored in aliquots. The concentration was determined by Bradford protein assay (Thermo Fisher Scientific) with BSA as standard. The protein purity and molecular weight were determined by standard SDS-PAGE along with Western blot confirmation using a rabbit anti-GST polyclonal Ab (GenScript, A00097). Recombinant GST-IdeZ was stored in 50 mM Tris-HCl, 150 mM NaCl, and 10% glycerol, pH 8.0. Endotoxin was removed from recombinant protein using High Capacity Endotoxin Removal Spin Columns (Thermo Fisher Scientific catalog 88274) following the manufacturer’s instructions.

### SDS-PAGE and analysis of IdeZ enzyme activity.

Pooled human IgG was purchased from MilliporeSigma (I4506); mouse and dog serum samples were obtained from in-house lab stocks or gifts from David Mack (University of Washington, Seattle, Washington, USA). Individual human serum samples from donors were purchased from Valley Biomedical. Rhesus macaque sera were gifts from Alice Tarantal (UCD, Davis, California, USA), Yoland Smith (Emory University School of Medicine, Atlanta, Georgia, USA), and Adriana Galvan (Yerkes National Primate Center, Emory University, Atlanta, Georgia, USA). Proteins analyzed by SDS-PAGE were separated under reducing conditions on NuPAGE 4–12% Bis-Tris (Invitrogen, Thermo Fisher Scientific) or on Mini-Protean TGX 4%–15% gels (Bio-Rad) and stained with Coomassie blue. All in vitro activity assays with recombinant GST-IdeZ or IdeZ (New England Biolabs catalog P0770S) (1 μg/reaction) were performed for 3 hours at 37°C, and serum samples were diluted 50 times in PBS before analysis by SDS-PAGE. All in vivo activity assays were performed with recombinant GST-IdeZ with mouse or NHP serum samples being diluted 10 times in PBS before analysis by SDS-PAGE and immunoblotting. Digested sera were probed with rabbit anti–human IgG-HRP H+L secondary antibody (Thermo Fisher Scientific catalog A18903, 1:10,000), rabbit anti–human IgG Fc HRP secondary antibody (Thermo Fisher Scientific catalog 31423, 1:10,000), and rabbit anti–human IgG F(ab′)_2_ HRP secondary antibody (Thermo Fisher Scientific catalog 31482, 1:10,000).

### Cell lines and recombinant virus production.

HEK293 cells (University of North Carolina Vector Core) were maintained in DMEM supplemented with 10% FBS, 100 U/mL penicillin, and 100 μg/mL streptomycin. Cells were maintained in 5% CO_2_ at 37°C. Recombinant AAV vectors were generated using triple plasmid transfection with the AAV Rep-Cap plasmid (pXR8 or pXR9 encoding AAV8 or AAV9 capsid proteins, respectively), adenoviral helper plasmid (pXX680), and a luciferase transgene cassette driven by the CBA promoter (pTR-CBA-Luc), flanked by AAV2 inverted terminal repeat (ITR) sequences. Viral vectors were harvested from media and purified via iodixanol density gradient ultracentrifugation followed by PBS buffer exchange. Titers of purified virus preparations were determined by quantitative PCR using a Roche Lightcycler 480 (Roche Applied Sciences) with primers amplifying the AAV2 ITR regions (forward, 5′-AACATGCTACGCAGAGAGGGAGTGG-3′; reverse, 5′-CATGAGACAAGGAACCCCTAGTGATGGAG-3′) (IDT Technologies).

### In vitro antibody and serum neutralization assays.

Pooled human IgG (25 μg undiluted) or antiserum (25 μL) (as specified for individual experiments) was mixed with an equal volume containing recombinant AAV9-Luc vector (100,000 vg/cell) in tissue culture–treated, black, glass-bottom, 96-well plates (Corning) and then incubated at room temperature for 30 minutes. For neutralization assays, 1 μg of GST-IdeZ was incubated with pooled human IgG in 5% CO_2_ at 37°C for 2 hours before addition of AAV9-CBA-Luc vector. A total of 1 × 10^4^ HEK293 cells in 50 μL DMEM + 10% FBS + penicillin-streptomycin was then added to each well, and the plates were incubated in 5% CO_2_ at 37°C for 24 hours. Cells were then lysed with 25 μL of 1× passive lysis buffer (Promega) for 30 minutes at room temperature. Luciferase activity was measured on a VICTOR3 multilabel plate reader (PerkinElmer) immediately after the addition of 25 μL of luciferin (Promega). All readouts were normalized to controls with no antibody/antiserum treatment. Recombinant AAV vectors packaging CBA-Luc transgenes, antibodies, sera, and GST-IdeZ were prediluted in DMEM and used in this assay.

### Mouse studies.

All animal experiments were performed using 6- to 8-week-old male and female C57BL/6 mice purchased from The Jackson Laboratory. Mice were injected IP with pooled human IgG (8 mg). The same mice were injected IV 24 hours later with PBS or recombinant GST-IdeZ (2.5 mg/kg). Recombinant AAV9-CBA-Luc or 1× PBS (as mock treatment) was injected 72 hours post–IdeZ treatment at a dose of 1 × 10^13^ vg/kg. Luciferase transgene expression levels were analyzed 4 weeks postinjection in the liver, heart, and kidney. Animals were sacrificed 4 weeks post–AAV9 injection with an IP injection of tribromoethanol (Avertin) (0.2 mL of 1.25% solution) followed by transcardial perfusion with 3 mL of 1× PBS. For human serum samples/IdeZ studies, 2 mice were injected IP with 200 μL human sera (purchased from Valley Biomedical, gift from StrideBio, Inc., Durham, North Carolina, USA). The same mice were then injected intravenously 72 hours later with PBS or recombinant GST-IdeZ (2.5 mg/kg). Mice were subsequently injected IV 72 hours post–IdeZ treatment with AAV9-Luc (1 × 10^13^ vg/kg).

### NHP studies.

A total of 3 cynomolgus macaques (3 males) designated for use in this study were obtained from Southern Research, who obtained them from Worldwide Primates, Inc. Animals were acclimated before study start and deemed healthy before study initiation. On the first day of dosing, the animals were approximately 3 years of age, were of male sex, and weighed between 2.4 and 3.8 kg. Animals were tested for preexisting AAV9 NAbs using an in-house in vitro NAb assay and were designated as M16561 being seronegative, M16556 being seropositive, and M16558 being seropositive. The seropositive NHP M16558 was administered IdeZ (0.5 mg/kg) via IV bolus injection on day 0. AAV9-CBA-Luc was administered to all 3 NHPs via IV bolus injection, 72 hours post–IdeZ injection at a dose of 5 × 10^12^ vg/kg. All animals had blood collected for analysis on days 0, 3, and 28. On day 28, NHPs were euthanized and organs collected via whole-body perfusion with sterile saline while under anesthesia following collection of specified blood samples.

### Tissue analysis for luciferase expression.

To quantify luciferase expression, animals injected with AAV9-CBA-Luc transgene were sacrificed, and tissues were harvested and frozen at 80°C. Tissues were later thawed, weighed, and lysed by adding 200 μL of 1× passive lysis buffer (Promega) before mechanical lysis using a Tissue Lyser II 352 instrument (QIAGEN), followed by centrifugation (21,100*g*, 2 minutes, 4°C) to remove any remaining tissue debris. To measure luciferase transgene expression, 15 μL of supernatant from each lysate was then loaded onto an assay plate along with 45 μL of luciferin, and luminometric analysis was performed using a VICTOR3 luminometer (PerkinElmer). The relative luminescence units obtained for each sample were then normalized to the input tissue weight for each sample, measured in grams, followed by log transformation.

### Tissue analysis for vector genome biodistribution.

DNA was extracted and purified from tissues using a QIAamp DNA FFPE Tissue Kit (catalog 56404; QIAGEN). Viral genome copy numbers were then determined for each tissue using quantitative PCR with primers specific to the CBA promoter (forward, 5′-TGTTCCCATAGTAACGCCAA-3′; reverse, 5′-TGCCAAGTAGGAAAGTCCCAT-3′). These viral genome copy numbers were then normalized to the level of the mouse lamin B2 housekeeping gene using specific primers (forward, 5′-GGACCCAAGGACTACCTCAAGGG-3′; reverse, 5′-AGGGCACCTCCATCTCGGAAAC-3′). The biodistribution of viral genomes is represented as the ratio of vector genomes per cell or as vector genomes per nanograms of DNA extracted, followed by log transformation.

### Statistics.

Where appropriate, data are represented as mean or mean ± standard deviation. Where appropriate, data were log transformed before statistical analysis. For data sets with at least 3 groups, significance was determined by 1-way ANOVA, with Tukey’s posttest. For analysis of the human sera data, significance was determined by the nonparametric Mann-Whitney rank test. *P < 0.05, **P < 0.01, ***P < 0.001, and ****P < 0.0001.

### Study approval.

All mice were maintained and treated in compliance with NIH guidelines and as approved by the Duke University Institutional Animal Care and Use Committee. Housing and animal care for NHP studies conformed to the guidelines of the US Department of Agriculture (Animal Welfare Act; Public Law 99-198) and those of the *Guide for the Care and Use of Laboratory Animals* (National Academies Press, 2011) and to the applicable Standard Operating Procedures at Southern Research.

## Author contributions

ZCE and AA designed all experiments, interpreted the data, and wrote the manuscript. ZCE and DKO carried out all molecular biology, virus production, and neutralization studies. KES and MMF carried out animal studies and assisted with tissue analysis.

## Supplementary Material

Supplemental data

## Version 1 09/17/2020

In-Press Preview

## Version 2 10/02/2020

Electronic publication
