# Supplementary material for: Rescuing AAV gene transfer from neutralizing antibodies with an IgG-degrading enzyme
Source: JCI Insight. 2020 Sep 17;5(19):e139881. doi: 10.1172/jci.insight.139881 (PMC7566709; doi:10.1172/jci.insight.139881)
Supplement: Supplemental data [file jciinsight-5-139881-s116.pdf]

Supplementary Information

Rescuing AAV gene transfer from neutralizing antibodies with an IgG-degrading enzyme

Zachary C. Elmore[1], Daniel K. Oh[1], Katherine E. Simon[1], Marco M. Fanous[1], Aravind Asokan[1,2,3,4*]

[1]Department of Surgery, [2]Department of Molecular Genetics & Microbiology, Duke University School of Medicine, Durham, NC, USA, [3]Department of Biomedical Engineering, [4]Regeneration Next, Duke University, Durham, NC,USA

*Corresponding Author: Aravind Asokan, Ph.D.

5148, MSRB3,

3 Genome Court,

Durham NC 27710, USA

Email: aravind.asokan@duke.edu

Supplementary Figures

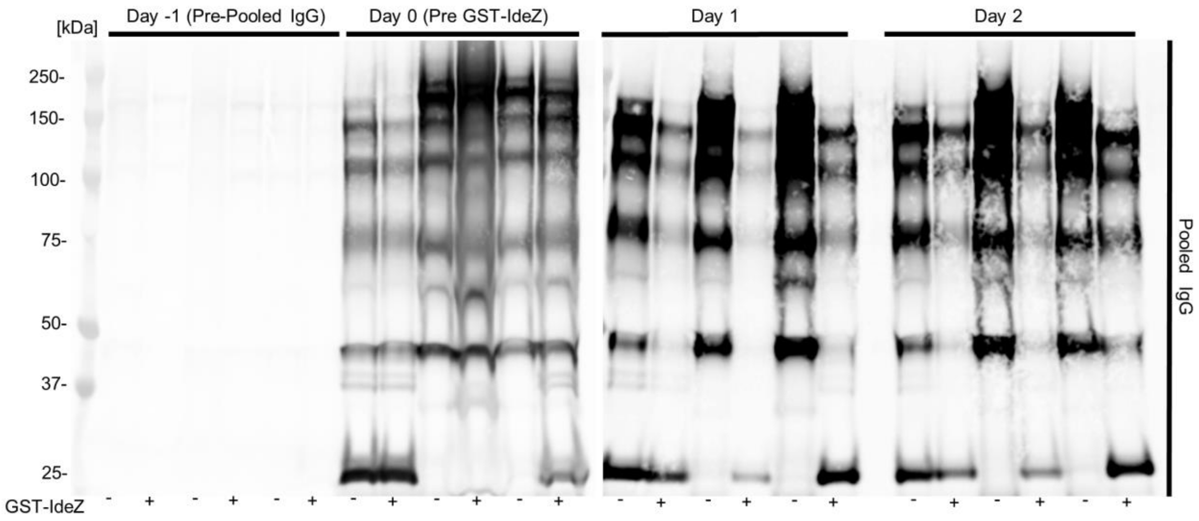

Supplemental Fig 1. Kinetic analysis of IdeZ mediated cleavage of pooled human IgG *in vivo*. Mice were injected intraperitoneally with pooled human IgG. The same mice were injected intravenously 24 hours later with PBS (-) or recombinant GST-IdeZ (2.5 mg/kg) (+). Blood samples were taken 24 and 48 hours post injection and analyzed by SDS-PAGE under reducing conditions and probed with human IgG specific antibodies to analyze IgG cleavage.

A.

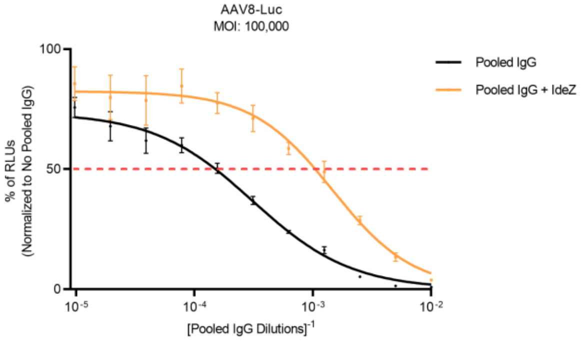

B.

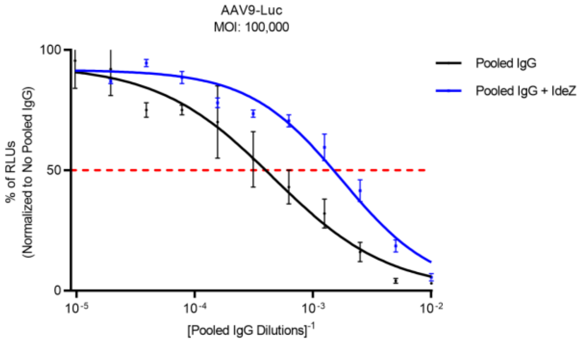

Supplemental Fig 2. IdeZ mitigates human IgG mediated neutralization of AAV8 and AAV9 *in vitro*.

Pooled human IgG was treated with PBS or GST-IdeZ (1ug). Samples were then serially diluted in twofold increments from 1:100 to 1:102,400 and then co-incubated with AAV8-Luc (**A**), or AAV9-Luc (**B**) *in vitro* (100,000 vg/cell). The dotted red line represents NAb-mediated inhibition of AAV8/9 transduction by 50%. Solid lines represent relative transduction efficiencies of AAV8/9 incubated with IgG (black) and AAV8/9 incubated with IgG treated with GST-IdeZ (yellow,blue) at different dilutions. Error bars represent SEM (AAV8, n = 3) (AAV9, n=2).

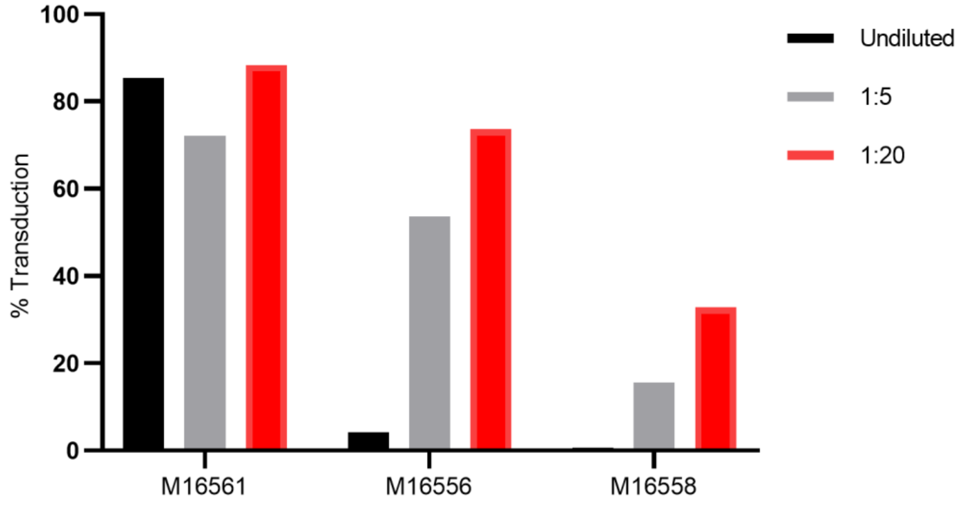

Supplemental Fig 3. AAV9 neutralization profile of NHP sera. NHP sera from three individual animals (M16561, M16556, M16558) were diluted from 1:5 to 1:20 and then coincubated with AAV9-Luc *in vitro* (100,000 vg/cell). Transduction levels were normalized to no serum control.

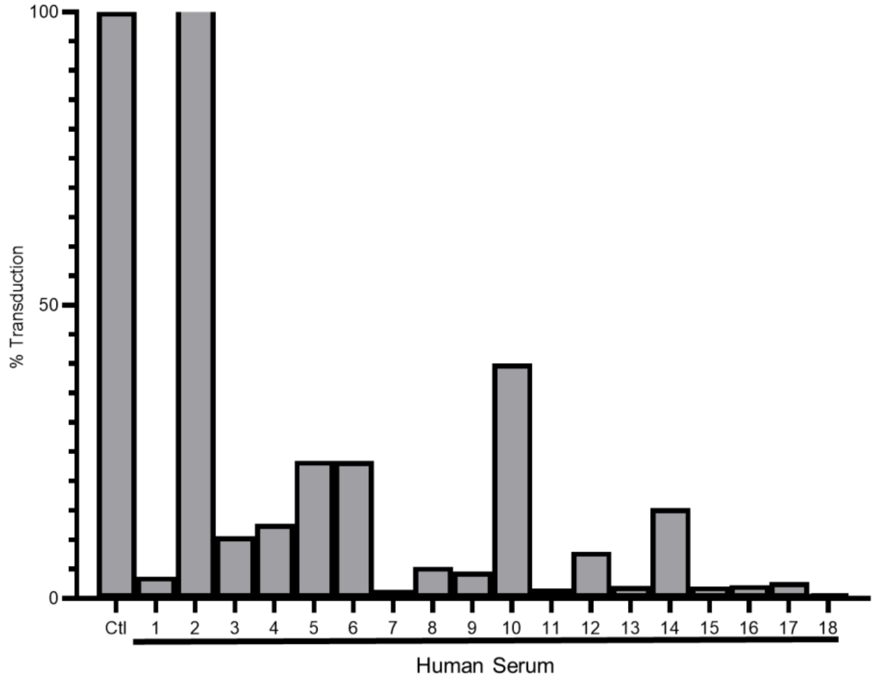

Supplemental Fig 4. AAV9 neutralization profile of human sera. Human sera from 18 individuals was diluted 1:5 and then coincubated with AAV9-Luc *in vitro* (100,000 vg/cell). Transduction levels were normalized to no serum control. Luciferase activity was determined at 24 hrs post-transduction. Detailed methods are described in Tse *et al.*, PNAS, 2017.

Supplemental Table 1. Demographic analysis of human serum used in this study.

Serum #	Lot #	Gender	Age	Race
**1**	BRH1499625	Male	37	Hispanic
**2**	BRH1499636	Male	32	Black
**3**	BRH1499659	Female	22	Black
**4**	BRH1499617	Male	32	Black
**5**	BRH1499665	Female	35	Hispanic
**6**	BRH1499637	Male	37	Caucasian
**7**	BRH1536076	Male	11	Caucasian
**8**	BRH1499666	Female	27	Black
**9**	BRH1499685	Female	35	Caucasian
**10**	BRH1499676	Female	35	Caucasian
**11**	BRH1499638	Male	30	Caucasian
**12**	BRH1499620	Male	21	Hispanic
**13**	BRH1536073	Female	16	Black
**14**	BRH1499645	Male	34	Caucasian
**15**	BRH1499679	Female	33	Caucasian
**16**	BRH1499647	Male	35	Caucasian
**17**	BRH1499669	Female	23	Hispanic
**18**	BRH1499660	Female	35	Hispanic
